# Single‐cell genomics meets systems neuroscience: Insights from mapping the brain circuitry of stress

**DOI:** 10.1111/jne.70005

**Published:** 2025-02-16

**Authors:** Naresh K. Hanchate

**Affiliations:** ^1^ Genetics & Genomic Medicine Department UCL Great Ormond Street Institute of Child Health, University College London London UK

**Keywords:** neural circuits, single‐cell transcriptomics, stress

## Abstract

Responses to external and internal dangers is essential for survival and homeostatic regulation. Hypothalamic corticotropin‐releasing hormone neurons (CRHNs) play a pivotal role in regulating neuroendocrine responses to fear and stress. In recent years, the application of neurogenetic tools, such as fiber photometry, chemogenetics and optogenetics, have provided new insights into the dynamic neuronal responses of CRHNs during stressful events, offering new perspectives into their functional significance in mediating neurobehavioural responses to stress. Transsynaptic viral tracers have facilitated the comprehensive mapping of neuronal inputs to CRHNs. Furthermore, the development and application of innovative single‐cell genomic tools combined with viral tracing have begun to pave the way for a deeper understanding of the transcriptional profiles of neural circuit components, enabling molecular‐anatomical circuit mapping. Here, I will discuss how these systems neuroscience approaches and novel single‐cell genomic methods are advancing the molecular and functional mapping of stress neurocircuits, their associated challenges and future directions.

## 1 INTRODUCTION

Mental health disorders are one of the biggest challenges in modern societies and a leading cause of disability worldwide.^1^ Depression alone affects around 300 million individuals worldwide. Recent studies estimate the prevalence of mental illnesses is rapidly rising at alarming rates, particularly among adolescents and young individuals.^2^ Furthermore, the prevalence of neurodevelopmental disorders, such as autism, which are commonly associated with stress‐related disorders, are also on the rise globally.^3^ It is of urgent need to fully understand the neurobiological pathways regulating stress.

In response to danger or stress, the brain activates several regulatory pathways to mediate appropriate behavioural and physiological responses essential for survival.^4^, ^5^, ^6^ The hypothalamus‐pituitary‐adrenal (HPA) axis is a pivotal component of this response.^4^ Within the hypothalamus, a subset of neurons in the paraventricular nucleus of the hypothalamus (PVN) that produce corticotropin‐releasing hormone or CRH (PVN^CRHNs^) control blood levels of stress hormones.^7^, ^8^, ^9^ Stress‐induced activation of PVN^CRHNs^ results in CRH release into the hypophyseal portal system at the median eminence. CRH then stimulates the anterior pituitary to release adrenocorticotropic hormone (ACTH), which in turn acts on the adrenal glands to increase blood levels of glucocorticoids (cortisol in humans and corticosterone in rodents). Elevated stress hormones, in turn, act on multiple tissue systems, such as increases in blood pressure, blood glucose, and respiratory effort, to coordinate appropriate responses to threats. After the stressor has ended, stress hormones convey negative feedback to the pituitary and brain to terminate the stress response. However, repeated and prolonged exposure to stress can heighten the risk of developing a wide variety of disorders, including neuropsychiatric (such as anxiety and depression), metabolic (such as obesity and diabetes) and cardiovascular.^10^

PVN^CRHNs^ play a crucial role in integrating information on a wide variety of stressors, including exteroceptive (sensory) and interoceptive (homeostatic and metabolic) signals, and perceived ‘psychogenic’ stressors (innate or acquired ones based on experiences). Decades of research have made significant advances in our understanding of the brain structures involved in processing fear and stress. Using traditional neuroanatomical tracers and genetically modified viral tracers, these efforts have generated a ‘physical’ map of brain circuitry that provide direct and indirect inputs to PVN^CRHNs^. These include neural inputs from areas within the hypothalamus, and several other brain areas, including the amygdala, hippocampus, bed nucleus of stria terminalis, lateral septum and brain stem.^4^, ^5^, ^11^, ^12^, ^13^, ^14^

With the advent of single‐cell and spatial genomic technologies, it is now possible to comprehensively generate molecular cell atlases of specific brain areas or the entire brain of small and large model organisms.^15^, ^16^, ^17^, ^18^ In more recent years, coupling single‐cell genomic tools with transsynaptic pseudorabies^19^ and monosynaptic rabies^20^ viral tracers has made it possible for the transcriptional profiling of circuit components that feed inputs to genetically defined neuronal cell types in specific brain areas in vivo^19^, ^20^, ^21^ or in vitro.^22^ In this review, I will provide an overview of recent studies employing advanced opto‐ and chemogenetics demonstrating the role of PVN^CRHNs^ in regulating stress‐related behaviours, reshaping the classical view of mere neuroendocrine populations. The application of viral tracers were crucial in deconstructing an anatomical, molecular, and functional map of mono‐ and polysynaptic inputs to PVN^CRHNs^. Single‐cell RNA‐sequencing emerged as powerful tools for resolving the transcriptional heterogeneity of PVN^CRHNs^. Finally, although still in their infancy, the development and application of novel methods (e.g., Connect‐seq) are facilitating the transcriptional profiling of neurons upstream of PVN^CRHNs^.

## 2 PVN^CRHNs^ GOVERN NEUROENDOCRINE AND BEHAVIOURAL RESPONSES TO STRESS

**FIGURE 1:**
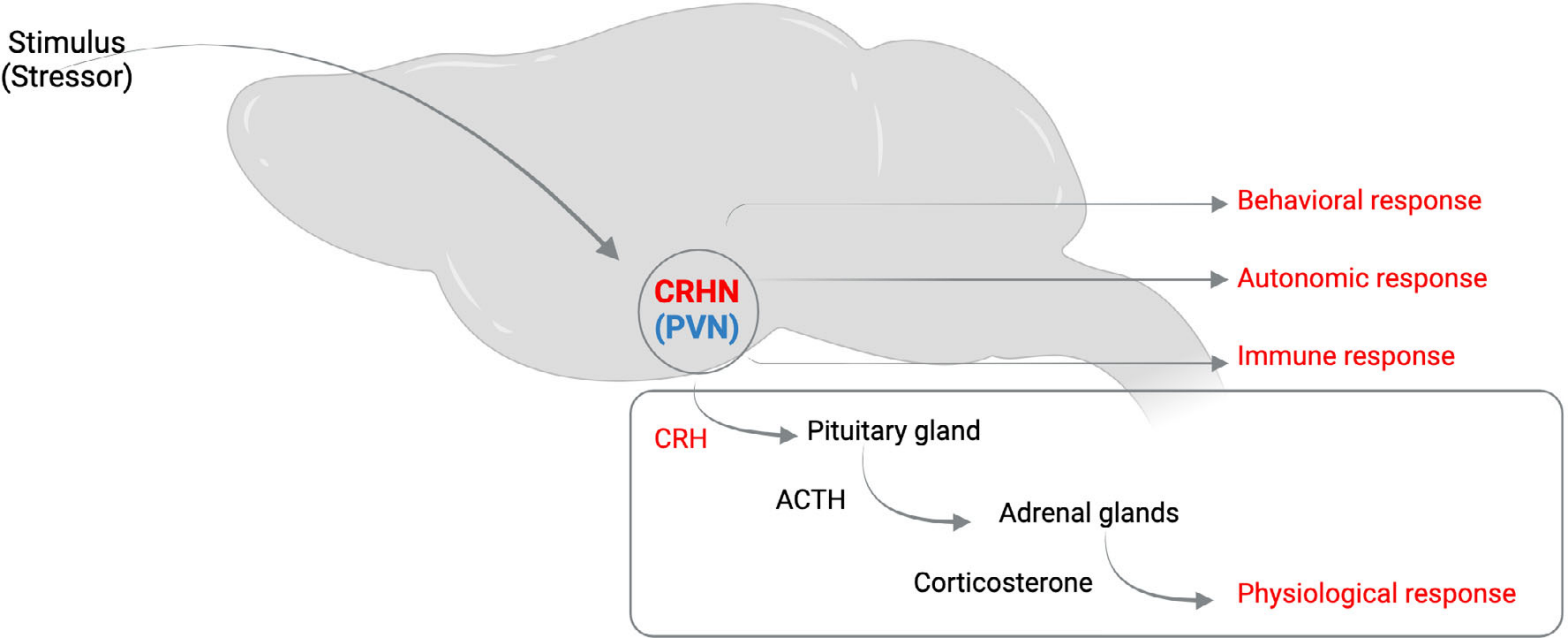
PVN^CRHNs^ are a crucial component of stress response pathways. A schematic illustrating diverse functional roles of PVN^CRHNs^ in eliciting behavioural, autonomic, immune and physiological responses to a wide variety of external (exteroceptive), internal (interoceptive), social (psychosocial), metabolic and physical stressors. The HPA axis, via the glucocorticoids, can in turn act on multiple organ systems to coordinate appropriate stress responses.

CRH, a 41 amino acid peptide hormone, was identified in 1981 as the hypothalamic stimulator of pituitary corticotropin (ACTH).^23^ Since the discovery of CRH, enormous progress has been made in the field of neuroendocrinology of stress. CRH emerged as one of the critical signalling molecules implicated in the regulation of stress and stress‐related mental health disorders.^4^, ^8^, ^24^ While CRH is widely expressed in subsets of neurons in different brain areas (See Allen Brain Atlas^25^), including the olfactory bulb, olfactory piriform cortex, BNST (bed nucleus of stria terminalis), central amygdala, hippocampus, cerebral cortex, cerebellum, and a few areas within the hypothalamus,^25^, ^26^, ^27^, ^28^ the PVN^CRHNs^ emerged as master regulators of the HPA axis in mediating physiological responses to fear and stress (Figure 1). Thereby PVN^CRHNs^ provide an entry point to genetically access the brain's stress neurocircuits.

Using conventional histological methods to detect expression of neuronal activity‐regulated genes, also called immediate early genes (IEGs), such as *Fos*, *Egr1*, and *Arc*, numerous studies have demonstrated that various internal and external dangers (or stressors) readily activate PVN^CRHNs^. These include immobilisation,^29^, ^30^ electric foot shock, noxious mechanical stimulus (pain),^30^ seizures,^31^ hypertonic solution,^32^ nociceptive activation,^33^ physical restraint,^34^ and forced swim.^35^

This body of research using conventional techniques highlights that the PVN^CRHNs^ are activated by a range of aversive stimuli, and they constitute the final crucial pathway through which the brain regulates physiological stress responses. However, using IEGs to map neuronal responses have limitations. These include (1) they provide a snapshot of neuronal response at a specific time point in post‐mortem tissues; (2) they lack the temporal resolution of neuronal activity dynamics of PVN^CRHNs^ in live animals during stressful experiences; and, most importantly, (3) IEGs cannot be used to study the dynamics of neuronal responses of PVN^CRHNs^ to neutral or attractive stimuli, or a combination of multiple stimuli.

These limitations have been recently overcome with the application of fiber photometry to study the neuronal activation dynamics of PVN^CRHNs^ in freely moving animals in different physiological and stressful contexts. Fiber photometry is an optical imaging technique that enables measurements of neuronal activity‐dependent calcium changes of genetically defined cell types within a specific brain area in freely moving animals.^36^, ^37^ The application of fiber photometry revealed that PVN^CRHNs^ are rapidly activated in response to a variety of aversive external stimuli. These include external sensory modalities such as olfactory predator odour cues (e.g., 2,3,5‐Trimethyl‐3‐thiazoline [TMT] or cat fur), visual cues (e.g., bright light or a looming disk that mimics a predator),^38^ and auditory (e.g., loud noises).^39^, ^40^ PVN^CRHNs^ are also activated by internal homeostatic and metabolic stressors, such as hunger, thirst, loss of blood, and changes in blood osmolarity, and by psychosocial stressors, such as social confrontation between a resident male and an intruder male,^39^ or during wakefulness.^41^ In contrast, appetitive stimuli, such as food odour^39^ or reward^42^ (e.g., sucrose), readily suppress PVN^CRHNs^ neuronal activity.

The rapid activation or inhibition of PVN^CRHNs^ in freely moving animals during natural behaviours at sub‐second timescales suggested that PVN^CRHNs^ might be involved in regulating stress‐induced behavioural responses as the neuroendocrine activation of the HPA axis has a much slower response of several minutes to hours to elevate blood levels of stress hormones. Several studies tested these hypotheses using optogenetics and chemogenetics to selectively activate or inhibit PVN^CRHNs^ and measure behavioural changes. Application of these tools revealed that the PVN^CRHNs^ orchestrate distinct and temporally organised complex behaviours,^43^ such as grooming, rearing and walking, or selection of aversive, attractive^38^ or defensive behaviours during stressful events.^39^ These studies indicate that PVN^CRHNs^ are not mere ‘effector’ neuroendocrine cells implicated in the regulation of stress hormones but are essential components of hypothalamic circuits that rapidly integrate a range of internal and environmental stimuli to elicit a dynamic and fitting behavioural response to stress followed by a slower neuroendocrine response.

## 3 DIRECT NEURONAL INPUTS TO PVN^CRHNs^

Given the biological significance of hypothalamic PVN^CRHNs^ in regulating stress responses, it has been critical to map the neuronal networks to better understand how stress signals are integrated, processed, and transmitted to PVN^CRHNs^. Studies during the 1980s and 1990s by Larry W. Swanson and Paul E. Sawchenko and several others mapped the neuroanatomical organisation of the hypothalamus, including the neuroendocrine systems, and the PVN neuronal inputs and outputs (for detailed reviews, see References 4, 9, 11, 12, 13, 14). These studies employed a range of first‐generation anterograde and retrograde axonal tract tracers to map neuronal inputs to the PVN and their outputs to downstream brain areas. For instance, retrograde axonal transporters (such as True Blue,^44^ Fluorogold^45^ or Cholera Toxin B^46^) were injected into the PVN to retrogradely label cells projecting from different brain areas. Similarly, anterograde tracers (such as Phaseolus vulgaris leucoagglutinin [PHA‐L]^47^) were used to map the downstream projections of neurons in the PVN

These studies have provided a detailed map of neuronal inputs and outputs to the PVN (summarised below). However, conventional tracers have several limitations: (1) a lack of neuronal specificity as the tracers can be uptaken non‐selectively by the neurons at the injection sites, thereby do not permit selective targeting of neurons defined by molecular or genetic markers; (2) uptake of tracers by axons of passage, thereby mislabelling cells; and (3) lack of transsynaptic transport, thereby making it challenging to map indirect pathways that are two or more synapses away.

In contrast to conventional tracers, viral vectors have been indispensable tools in neuroscience research, particularly for circuit tracing.^48^ They can be genetically engineered based on promoters, enhancers, or Cre‐recombinase systems to selectively target specific cell types or their projections. Adeno‐associated viruses (AAVs) or lentiviruses are commonly employed for long‐term labelling with fluorescent proteins enabling the visualisation of cells. These viruses can also be engineered to carry genes encoding light‐sensitive proteins (channelrhodopsin or halorhodopsin) or engineered receptors (like DREADDs—Designer Receptors Exclusively Activated by Designer Drugs) for selective manipulations of cell types in a defined brain region using optogenetics^49^ or chemogenetics,^50^ respectively, or retrograde and anterograde tracing of neural circuits. In addition, modified versions of AAVs or Sindbis viruses are commonly used for anterograde tracing, and certain others, such as Canine Adenovirus Type 2 (CAV‐2), are used for retrograde labelling and studying long‐range projections in the brain. Furthermore, genetically modified versions of rabies virus (RVs) and pseudorabies viruses (PRVs) are commonly used for retrograde transneuronal tracing of directly or indirectly upstream neurons, respectively. PRVs offer several advantages in circuit tracing as they are self‐replicating and polysynaptic.^51^ Therefore, as PRVs travel across synapses from ‘starter’ cells to upstream neurons, they continue replicating, enabling their detection using immunolabelling methods.

**FIGURE 2:**
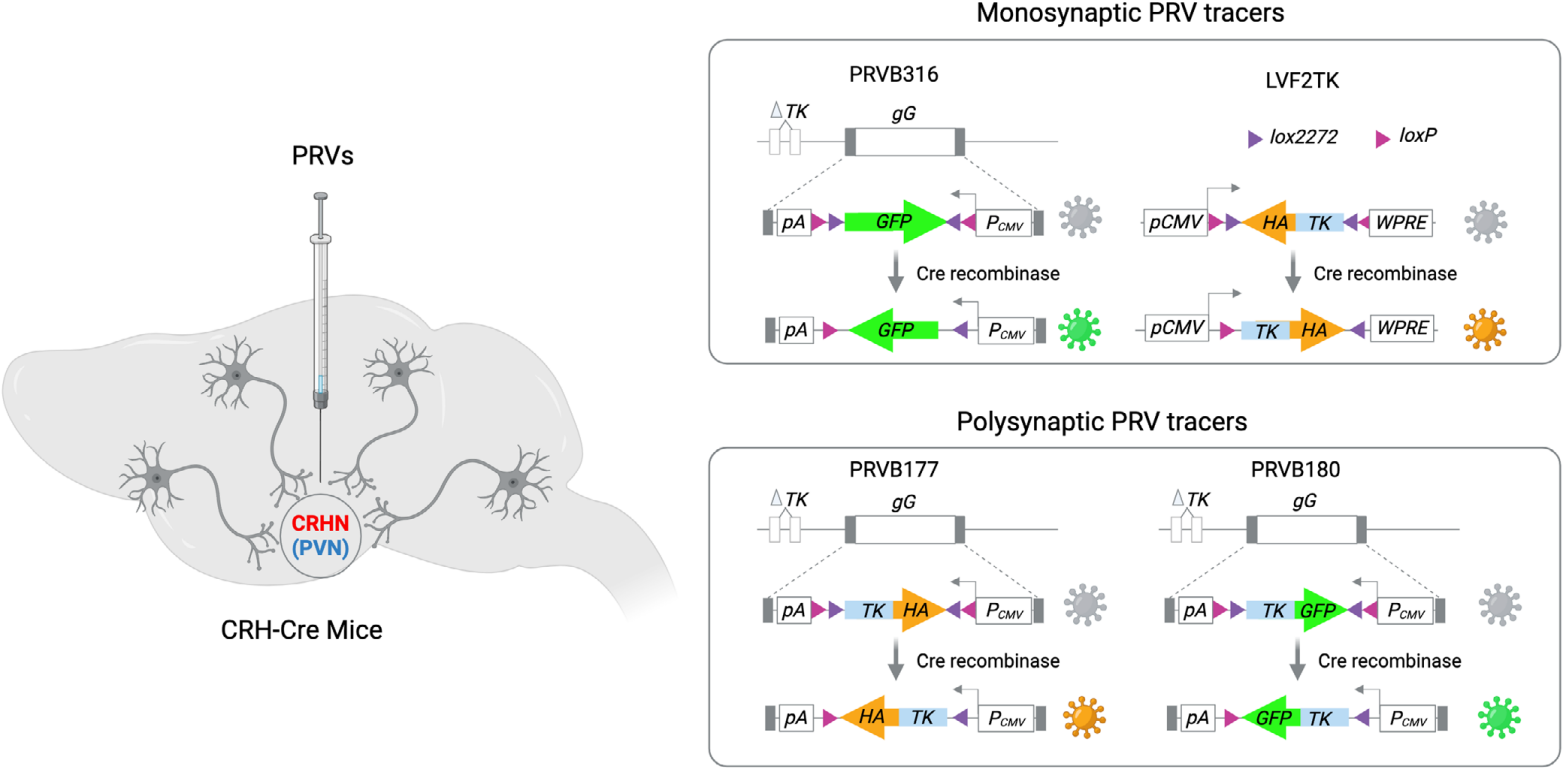
Neuroanatomical mapping of upstream neuronal circuitry using retrograde PRVs. [Left] Schematic illustrating stereotactic intracranial delivery of PRVs into the PVN of Crh‐Cre mice to infect CRHNs and trace upstream circuitry. [Right] PRVs are genetically modified to rely on thymidine kinase (TK) in a Cre‐dependent manner for their replication. Upon infection and replication of PRVs in CRHNs (Crh‐Cre mice), PRVs travel retrogradely to upstream neurons in a monosynaptic (PRVB316; TK provided by LV2TK) or polysynaptic (PRVB177/PRVB180) manner, respectively, and PRV‐infected cells can be mapped by immunolabelling brain sections with GFP (PRVB316/PRVB180) or HA (PRVB177) antibodies.

**FIGURE 3:**
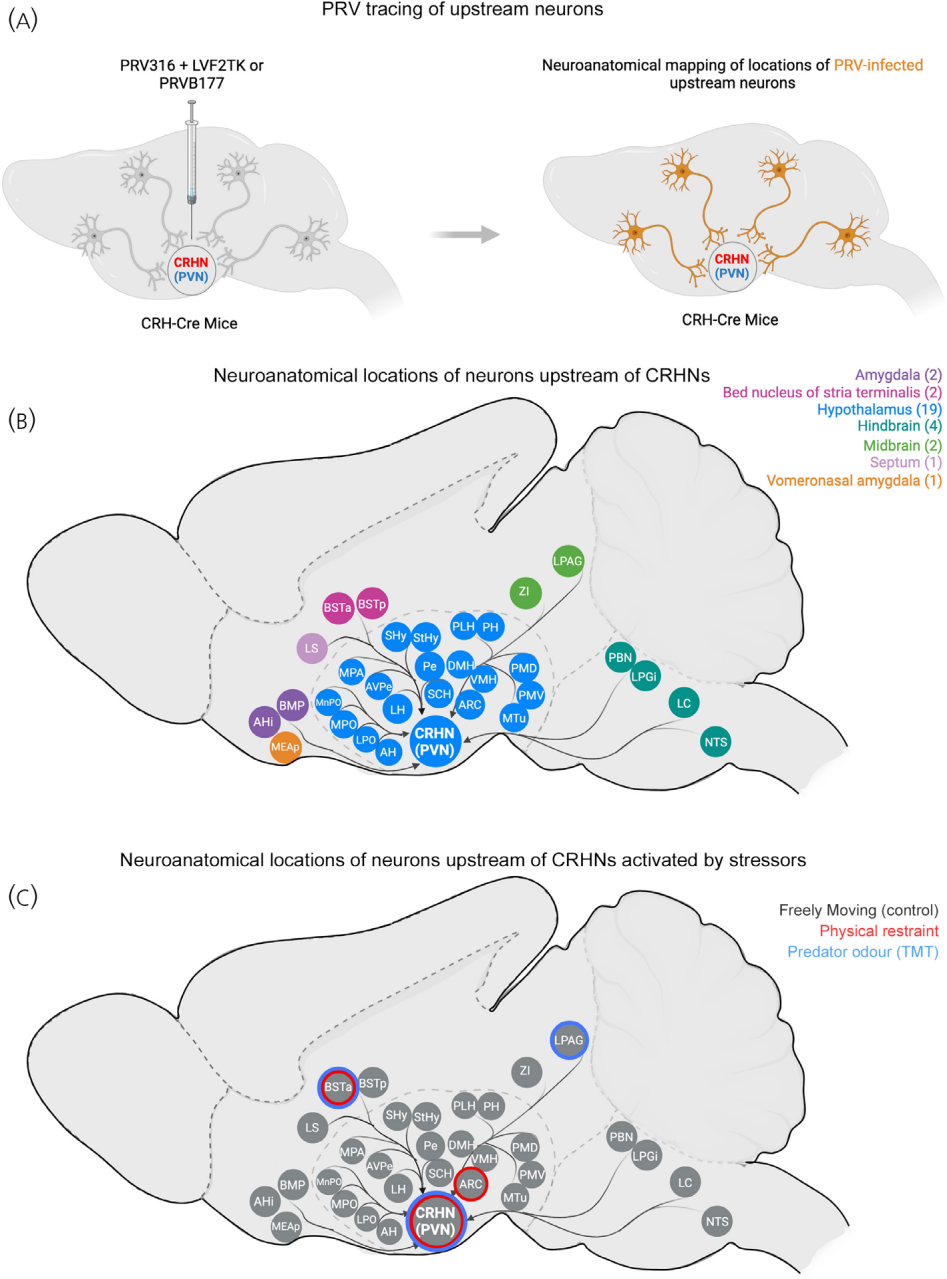
Stressor‐induced differential activation of CRHN neuronal circuity. (A) Schematic illustrating PRV delivery (co‐injection of PRVB316 + LVF2TK or PRVB177) into the PVN of CRH‐Cre mice to infect CRHNs. Brain sections from injected animals were then immunolabelled with GFP or HA antibodies to visualise virus‐infected cells across the brain. (B) Schematic illustrating a map of brain areas containing neurons that provide direct (monosynaptic) inputs to CRHNs. Brain areas contained within a broadly defined brain structures (e.g., hypothalamus in blue) are coloured similarly to distinguish between other neuroanatomical locations. (C) Stressors can be conveyed to CRHNs via parallel pathways. Schematic illustrating brain areas upstream of CRHNs activated by stressors (physical restraint in red and predator odour in blue). Neuronal activation of upstream neurons was detected in mice subjected to acute stressors versus control animals (freely moving) by dual labelling brain sections with HA (PRVB177) and riboprobes for neuronal activity marker *c‐Fos*.

Using Cre‐dependent pseudorabies viruses (PRVs), a recent study by Kondoh et al. mapped the brain areas that provide direct and indirect synaptic inputs to PVN^CRHNs^.^52^ They developed two strains of Cre‐recombinase‐dependent Bartha PRVs, PRVB316 and PRVB177, and a lentivirus (LVF2TK) with Cre‐dependent expression of haemagglutinin‐tagged Thymidine Kinase (HA–TK),^52^ which is required for PRV replication and spread across synapses (Figures 2 and 3A). Upon transduction of PVN^CRHNs^ (using Crh‐ires‐Cre mice) and Cre recombination, PRVB177 irreversibly expresses HA–TK that results in its replication and spread across multiple synapses (‘polysynaptic’ PRV). PRVB316 has a Cre‐dependent expression of GFP and TK is provided by the Lentivirus, LVF2TK. Thus, PRVB316 replicates only in PVN^CRHNs^ co‐infected with LVF2TK and spreads across one synapse, but not further, and is therefore a ‘monosynaptic’ PRV.

Retrograde viral tracing identified neurons directly upstream of PVN^CRHNs^ in 31 brain areas (Figure 3), including the hypothalamus, BNST, lateral septum, amygdala, and areas in the midbrain and hindbrain, and an additional 30 brain areas indirectly upstream of PVN^CRHNs^. In line with the previous findings, viral tracing confirmed robust synaptic inputs from most hypothalamic nuclei to PVN^CRHNs^. These include areas in the tuberal hypothalamus, dorsomedial hypothalamus (DMH), ventromedial hypothalamus (VMH), suprachiasmatic nucleus (SCh), and the arcuate nucleus of the hypothalamus (ARC), which are linked to autonomic regulation, aggression and reproductive behaviours, circadian rhythms, and energy homeostasis, respectively. Within the anterior regions, areas included the medial preoptic area (MPA), median preoptic nucleus (MnPO), lateral preoptic area (LPO), anteroventral periventricular nucleus (AVPe), and the anterior hypothalamic area (AH), which are implicated in thermoregulation, reproduction, cardiovascular responses, and other physiological functions. In addition to these, PRV tracing identified direct inputs from several areas in the posterior hypothalamus, including the peduncular part of the lateral hypothalamus (PLH), dorsal and ventral part of the premammillary nucleus (PMD and PMV), and the medial tuberal nucleus (MTu). Robust connectivity within the hypothalamus highlights PVN^CRHNs^ role in integrating diverse physiological inputs, including circadian rhythms, appetite and hunger, and reproduction, enabling effective regulation of the HPA axis.

The 12 extrahypothalamic areas that provide direct inputs to PVN^CRHNs^ were within the vomeronasal amygdala (the posterior part of medial amygdala, MEAp), the lateral septum (LS), the anterior and posterior parts of the bed nucleus of stria terminalis (BNSTa and BNSTp), two areas in the midbrain: zona incerta (ZI) & lateral periaqueductal grey (LPAG); and four areas in the hindbrain: parabrachial nucleus (PBN), lateral paragigantocellular nucleus (LPGi), nucleus of the solitary tract (NTS), and locus coeruleus (LC) (Figure 3B). These areas identified by PRV tracing are consistent with previous studies with a few exceptions (MEAp and LPGi). It was previously thought that the neurons in the MEAp send indirect projections to the PVN via the BNST, DMH and POA,^53^ but PRV tracing studies showed direct connections between MEAp to PVN^CRHNs^. Another finding from PRV tracing were direct synaptic connections from the LPGi to PVN^CRHNs^. LPGi is known for its roles in regulating blood pressure, REM sleep and autonomic functions. LS and BNST are well known brain structures that play crucial roles in fear and anxiety responses, and they act as major relay stations between the higher brain areas, including the amygdala, hippocampus and prefrontal cortex, and the PVN.^4^, ^11^, ^53^

These advancements highlight the versatility of viral tracers in resolving limitations of earlier methodologies. The findings underscore the complexity of PVN^CRHN^ circuits, with inputs integrating signals from diverse brain areas involved in homeostasis, autonomic regulation, and emotional processing. Future studies should leverage combinatorial viral approaches, such as integrating optogenetics, fiber photometry and single‐cell transcriptomics, to further dissect the molecular and functional diversity of these pathways.

## 4 INDIRECT NEURONAL INPUTS TO PVN^CRHNs^

Viral tracing have also identified indirect inputs from an additional 30 brain areas,^52^ including most brain structures previously associated with regulation of PVN^CRHNs^ neuronal activity. Indirectly upstream neurons were detected in multiple subregions of the amygdala, vomeronasal amygdala, hippocampus, olfactory areas, midbrain and hindbrain. Like with the findings on direct inputs, PRV tracing demonstrated parallels with prior results obtained through conventional tracing methods, while also shedding new insights into brain regions that provide indirect inputs to PVN^CRHNs^.

Notable findings from PRV tracing include a detailed characterisation of synaptic inputs from the olfactory cortical areas to the PVN^CRHNs^. Olfactory signals, such as predator odours, are well known to activate PVN^CRHNs^ to induce physiological stress responses, but the neural circuit pathways that mediate olfactory signals to PVN^CRHN^ had not been fully delineated. Volatile olfactory signals detected by the olfactory sensory neurons in the main olfactory epithelium are transmitted to the main olfactory bulb, whereas the peptides or proteins are detected by neurons in the vomeronasal organ and transmitted to the accessory olfactory bulb.^54^ From the main bulb, neurons project to different olfactory cortical areas, and from the accessory bulb, neurons project to the vomeronasal amygdala, BNST, and other brain regions. Signals are then sent to the PVN^CRHN^ via indirect pathways. As a result, conventional tracers may not fully capture these indirect connections between the olfactory cortical areas and the PVN, highlighting the need for transsynaptic tracers, like PRVs, in elucidating these pathways. PRV tracing revealed indirect synaptic inputs from five of nine olfactory cortical areas examined. These include the anterior cortical amygdala (ACo), amygdalo‐piriform transition area (AmPir), posterolateral cortical amygdala (PLCo), posterior part of the piriform cortex (pPir), and the lateral entorhinal cortex (LEnt). In the vomeronasal amygdala, PRV tracing identified direct inputs from MEAp and indirect inputs two subareas: MEAa and PMCo. Furthermore, PRV tracing identified indirect inputs from other cortical areas, including the medial entorhinal cortex (MEnt), caudomedial entorhinal cortex (CEnt), and the prefrontal cortex, which regulates negative‐feedback of the HPA‐axis via intermediary pathways known to project to the PVN.^55^

The hippocampus is well known to provide inhibitory inputs to PVN^CRHNs^ via indirect pathways.^4^ Anterograde tracing using phaseolus vulgaris‐leucoagglutinin (PHA‐L) has shown that the hippocampus projects to the BNST, amygdala, and other areas, but not directly to the PVN.^45^ Consistent with these results, PRV tracing revealed indirect connections between PVN^CRHNs^ and hippocampal regions, including neurons in the VS and CA1. Within the amygdala, PRV tracing revealed both direct and indirect synaptic inputs from various subnuclei. The amygdala, widely studied for its conserved role in regulating physiological and behavioural responses to fear, stress, and anxiety, consists of more than 13 subnuclei,^4^, ^56^ including the basolateral (BLA), central (CeA) and medial (MeA) nuclei. PRV tracing identified indirect inputs from the CeA, anterior part of basomedial amygdala (BMA), and anterior and posterior parts of basolateral amygdaloid nucleus (BLA and BLP). These findings are consistent with previous dual tract‐tracing analysis that showed limited direct connections between the CEA and the PVN.^57^ Conversely, the pMEA, posterior part of basomedial amygdala (BMP) and amygdalohippocampal area (AHi) provide direct synaptic inputs to PVN^CRHN^. These differences in pMEA to PVN connectivity may reflect variations in the amount of antigen (tracers transported in the axons vs. PRVs in upstream neurons) or transport mechanisms and antibody detection sensitivity, among other factors. Supporting these findings from PRV tracing, a recent optogenetics study demonstrated direct connections between the urocortin‐3 (Ucn‐3) neurons in the posterodorsal subnucleus of the medial amygdala (MePD) and the PVN.^58^ Optical activation of MePD Ucn‐3 projections in the PVN increased CORT secretion.

In the midbrain, PRV tracing detected neurons indirectly upstream of PVN^CRHNs^ in the substantia nigra (SNC), p1 periaqueductal grey (p1PAG), dorsal raphe nucleus (DRN), and the nucleus of the lateral lemniscus (NLL). In the hindbrain, consistent with earlier studies, PRV tracing identified direct inputs from the NTS, PBN, and LC areas. Beyond these regions, PRV tracing resolved indirect inputs from additional hindbrain subregions, including the laterodorsal tegmental nucleus (LDTg), subcoerulues nucleus (SubC), gigantocellular reticular nucleus (Gi), paraolivary nucleus (PO), lateral reticular nucleus (LRt) and intermediate reticular nucleus (IRt). These hindbrain regions may integrate stress‐related information and transmit signals to PVN^CRHNs^, either through neurons in the hindbrain or by projecting to neurons in other brain areas, contributing to the behavioural and physiological responses to stress.

These studies provide an in‐depth understanding of direct and indirect inputs to PVN^CRHNs^ to mediate stress responses, underscoring the complexity of stress regulating PVN^CRHNs^ neurocircuitry. A limitation of viral tracing is its inability to fully resolve the intermediate pathways linking neurons across multiple synapses away from PVN^CRHNs^. Future studies combining viral tracers with optogenetics, anterograde tracers, and fiber photometry could provide more precise mapping of these circuits.

## 5 STRESS‐INDUCED ACTIVATION OF PVN^CRHNs^ NEURONAL INPUTS

Neuroanatomical mapping studies have well‐established PVN^CRHNs^ activation in response to diverse stressors. In addition to the stressor‐induced PVN activation, numerous studies using immediate early gene (IEG) activation (such as c‐*Fos* and *Egr1*) have shown that stressors activate several other brain areas.^29^, ^30^, ^31^, ^32^, ^33^, ^34^, ^35^, ^59^ A subset of neurons within these areas are likely connected to the PVN and involved in transmitting signals to PVN^CRHNs^. These brain areas could also be involved in CRHN‐independent functions (e.g., to regulate memory, reproduction, or sleep). Further, the upstream neurons in the same brain area can contain genetically and functionally distinct neuronal subsets that can be differentially activated by various stimuli and be involved in transmitting different signals to PVN^CRHNs^.

Viral tracing provided a map of neuroanatomical locations of neurons upstream of PVN^CRHNs^ and laid the necessary foundation to map out the responses of PVN^CRHN^ circuitry. However, it remains to be fully characterised which of these upstream brain areas respond to particular stressors or stressor types, and which of these areas transmit excitatory or inhibitory signals to PVN^CRHNs^. To investigate these questions, viral tracing combined with analysis of neural activity markers (*Arc* and *c‐Fos*) have been used to identify and locate virus‐infected upstream neurons and map their responses to different stressors. Mice infected with PRVs were subjected to acute stressors and brain areas activated were identified by the expression of IEGs, *Arc* or *c‐Fos*, in PRV+ cells. PRV tracing studies revealed that the neurons in five olfactory cortical areas (pPir, ACo, PLCo LEnt and AmPir) provide indirect inputs (~two synapses away) to PVN^CRHNs^.^52^ Of the five cortical areas, acute exposure to two predator odours (Bobcat Urine and TMT), but not rabbit urine, significantly increased *nARC* in PRV+ cells in a single olfactory cortical area, amydalo‐piriform transition area (AmPir). Chemogenetic activation or silencing of AmPir resulted in a striking increase in stress hormone levels or blocked predator odour‐induced increases in stress hormone levels, respectively, without affecting the fear behaviour. These studies revealed that AmPir is vital in mediating innate endocrine response to predator scents. In another study, Lee et al. sought to identify the stimulus‐induced activation of neurons in the 31 brain areas that are directly upstream of PVN^CRHNs^.^60^ Acute exposure to the psychogenic stressors: physical restraint and predator odour (TMT), revealed that physical restraint activated PRV+ neurons in ARC and BNSTa, whereas predator odour activated upstream neurons in LPGi and BNSTa (Figure 3C).

Together, these studies suggest that a single olfactory cortical area can indirectly transmit predator odour signals to PVN^CRHNs^ via two synapses. Interestingly, studies by Lee et al. indicate that stressor signals can reach PVN^CRHNs^ via multiple parallel pathways; and that more than one area can convey a single stressor signal to PVN^CRHNs^, and finally a single brain area can transmit multiple stressor signals to PVN^CRHNs^.^60^

## 6 MOLECULAR HETEROGENEITY OF PVN^CRHNs^

The PVN, considered a hub of major neuroendocrine cells, plays a key role in coordinating neuroendocrine, autonomic, and behavioural responses. Based on a combination of tract tracing studies, Nissl‐based staining and the co‐expression of neuropeptides with neuroendocrine labelling methods, the PVN is divided into 10 distinct subnuclei within three major divisions: (1) magnocellular (PVHm) that contains neurons projecting directly to the posterior pituitary, (2) parvicellular (PVHp) that contains neuroendocrine neurons projecting to the median eminence and (3) the descending (PVHd) division with preautonomic neurons that project to the spinal cord and brain stem.^61^, ^62^ Studies in rats and mice showed differences in the spatial organisation of cell types and their functions in the PVN.^61^, ^62^ Overall, the rat PVN has a more distinct and compartmentalised architecture with distinct cellular organisations as compared to a less differentiated subdivisions in the mouse PVN. Furthermore, rat PVN shows clear separation between the magnocellular and parvicellular neuroendocrine neurons as compared to a less seggregated distribution in the mouse PVN.

The PVN^CRHNs^ are a heterogeneous population of cells broadly classified into two major subtypes: (1) neuroendocrine subpopulations, also the largest PVN^CRHNs^ subpopulation, send projections to the median eminence to regulate the HPA axis and (2) the preautonomic PVN^CRHNs^ send projections to the brain stem and spinal cord to regulate autonomic responses to stress (e.g., cardiovascular and respiratory functions).^4^, ^5^, ^9^, ^12^, ^13^ The neuroendocrine subpopulations also send axon collaterals to different brain areas to regulate behavioural responses to stress. The two PVN^CRHNs^ subpopulations exhibit species‐species differences in their neuroanatomical distribution within the PVN. In both rats and mice PVN, the majority of neuroendocrine PVN^CRHNs^ (~90% of total PVN^CRHNs^) are distributed in the dorsal zone of medial parvicellular part of PVN (PVNmpd) and a few others scattered in other subdivisions.^61^, ^62^, ^63^ However, their spatial distributions with surrounding magnocellular subpopulations are different in rats and mice PVN.

Subsets of PVN^CRHNs^ also coexpress a range of other neuropeptides. In rats, the magnocellular neurons expressing oxytocin (*Oxt*) and vasopressin (*Avp*), located in the magnocellular subdivision of the PVN co‐expressed small amounts of CRH,^11^, ^64^ which can increase up to ~70% following adrenalectomy.^65^ In mice, very few PVN^CRHNs^ (~3%–5%) co‐expressed *Oxt* or *Avp* under basal conditions.^63^ Subsets of PVN^CRHNs^ also coexpress a range of other neuropeptides in certain physiological states, including enkephalins (*Penk*),^66^ neurotensin (*Nts*), cholecystokinin (*Cck*), vasoactive intestinal peptide (*Vip*), galanin (*Gal*), dynorphins (*pDyn*) and angiotensin (*Agt*). However, not all neuropeptides are co‐expressed in the same PVN^CRHNs^. Instead, cells coexpress certain combinations of neuropeptides at different levels under certain physiological conditions.

For instance, adrenalectomy in rats induces an increase in co‐expression *Avp*, *Agt* and *Cck* in PVN^CRHNs^,^67^, ^68^ but not in basal conditions, whereas *Avp* levels were upregulated in PVN^CRHNs^ upon chronic stress.^69^ The co‐expression of AVP with CRH in the PVN allows for potent stimulation of pituitary corticotropes during chronic stress and fine‐tuning of the HPA axis response to stress.^69^, ^70^ The co‐expression of other neuropeptides (e.g., neurotensin, galanin) in PVN^CRHNs^ and their precise physiological roles are yet to be determined. This co‐regulation of CRH and AVP, and possibly other neuropeptides, is critical for the adaptive modulation of physiological and behavioural responses to stressors, ensuring that the body's response is appropriate for the nature and intensity of the stress encountered.^65^, ^66^, ^70^, ^71^ These observations clearly indicate high‐level heterogeneity of PVN^CRHNs^.

In recent years, single‐cell transcriptomics emerged as a powerful tool to further resolve molecular heterogeneity of PVN^CRHNs^. Several studies have transcriptionally profiled CRHNs in the hypothalamus or the PVN using different isolation methods.^60^, ^72^, ^73^, ^74^, ^75^, ^76^ Romanov et al. used chip‐based microfluidics to analyse 898 neurons from the mouse hypothalamus and reported four *Crh*+ cell clusters (a total of 192 cells; two populations of GABAergic *Crh* ^+/−^ that coexpress *Lhx6* or *Pgr15l* and two populations of glutamatergic *Crh*+ cells that are likely parvocellular from the PVN).^72^, ^73^, ^75^ Xu et al. used manual isolation methods using micropipette to isolate cells and analysed 706 single cells from the PVN and reported two glutamatergic clusters of PVN^CRHNs^ (total 119 cells) that co‐expressed *Scgn/Bcl11a* and *Ntng1*/*Fam150b*+.^74^ Single‐cell studies identifying PVN^CRHNs^ as predominantly glutamatergic are consistent with previous molecular characterisation of PVN^CRHNs^ using immunohistochemical studies.^77^ Chen et al. used Drop‐seq to analyse 3319 cells from the hypothalamus and reported four *Crh*+ cell clusters (two GABAergic and two glutamatergic).^78^ Lee et al. used manual isolation methods and analysed 18 *Crh*+ cells from the PVN and reported genes encoding receptors for neurotransmitters and neuropeptide ligands.^60^ More recently, Short et al. used flow cytometry to isolate fluorescently labelled *Crh*+ cells from the PVN and reported five clusters of *Crh+* cells (252 cells; three glutamatergic and two GABAergic) and reported early life adversity induces significant transcriptional changes in one of the glutamatergic clusters that coexpressed *Ntng1*.^76^ In addition to these, a more recent study by Berkhout et al integrated publicly available single‐cell RNA‐seq datasets on mouse PVN and reported a more detailed molecular subtypes within the PVN, including the molecular identification of preautonomic subtypes of PVN^CRHNs^ that coexpressed *Adarb2* and *Crh*.^79^

These studies demonstrated that PVN^CRHNs^ can be transcriptionally subclassified reflecting their diverse functional roles and their neuroanatomical locations in distinct PVN subnuclei. Interestingly, studies by Romanov et al. and Xu et al. revealed a subcluster of PVN^CRHNs^ that coexpressed *Scgn* (secretagogin), a calcium‐binding protein, which was then validated as the selective marker for the neuroendocrine subpopulation of PVN^CRHNs^. Using ultrastructural analysis and pharmacological tools, Romanov et al. further demonstrated that secretagogin regulates stress‐induced CRH release to activate the HPA axis, reporting a neuron‐specific calcium‐sensor selectively expressed in PVN^CRHNs^.^80^

## 7 SINGLE‐CELL TRANSCRIPTOMICS OF PVN^CRHNs^ NEURONAL INPUTS

Viral tracing provided a neuronal anatomical map of neuronal inputs to the PVN^CRHNs^. However, the map illustrates a partial picture of the circuitry as it lacks molecular information on the neuronal components, and the underlying signalling mechanisms of how they communicate with their downstream partners.^17^, ^81^, ^82^ The genetic identification and transcriptional profiling of circuit components is essential to understanding the underlying signalling mechanisms, to apply genetic tools to map their functional responses, and for investigating their physiological roles.^18^ In addition, brain areas sending inputs could potentially contain molecularly and functionally distinct subsets of upstream neurons, which could have different connectivity and functions.

**FIGURE 4:**
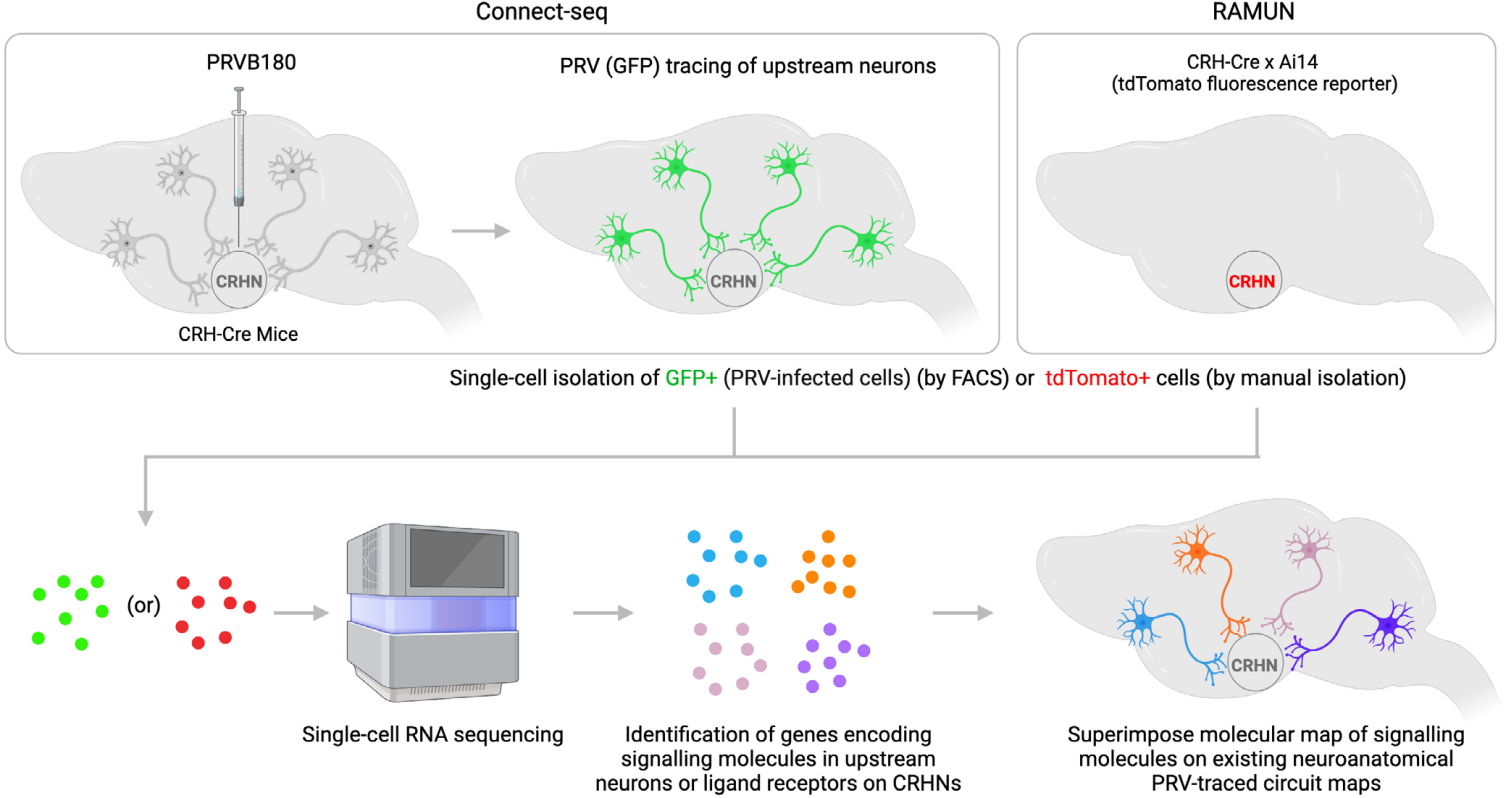
Single‐cell genomic strategies to molecularly map CRHN circuitry. Molecular identities of upstream neurons can be determined by two different single‐cell genomic strategies. In the first strategy, termed ‘Connect‐seq’, single‐cell transcriptomics of PRV‐infected cells were used to determine the genes encoding neurotransmitters or neuropeptides (neurochemical messengers). In the second strategy, termed ‘RAMUN’ (Receptor‐assisted Mapping of upstream neurons), single‐cell transcriptomics of CRHNs was used to determine the gene repertoire encoding receptors that bind to neurotransmitters or neuropeptides (neurochemical messengers) ligands. The neuroanatomical locations of ligands detected by Connect‐seq or RAMUN were then determined by dual labelling brain sections with riboprobes for the neuropeptide or neurotransmitter ligands and immunolabelling brain sections to detect PRV‐infected cells to generate a molecular map of CRHN brain circuitry.

To enable transcriptional profiling of neural circuit components, a new method, termed “Connect‐seq” has been developed. Connect‐seq couples single‐cell transcriptomics with retrograde viral tracing^19^ (Figure 4). It employs a new PRV strain (PRVB180)^19^ (Figure 2), which has Cre‐dependent expression of GFP fused to TK. Upon transduction of PVN^CRHNs^ in Crh‐Cre mice and Cre‐dependent recombination, PRVB180 replicates and spreads across synapses retrogradely. PRV‐infected upstream cells labelled with GFP can then be isolated using fluorescence‐activated cell sorting (FACS) and analysed by RNA sequencing to profile their transcriptomes.

**FIGURE 5:**
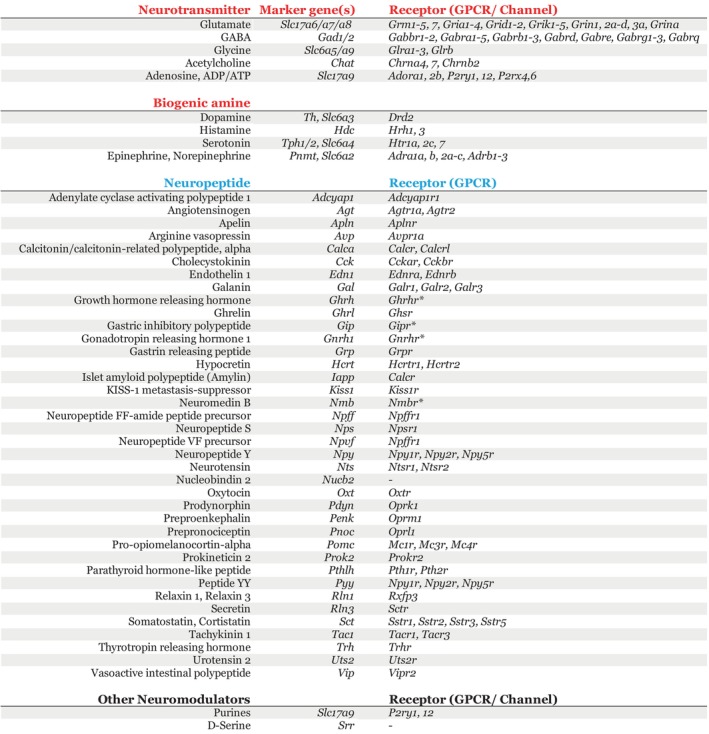
The ‘interactome’ of ligand‐receptors at CRHN synapses. Table illustrating the genes encoding neuronal signalling molecules (neurotransmitters, neuropeptides, biogenic amines, other neuromodulators) detected in presynaptic neurons using Connect‐seq. Upstream presynaptic neurons can employ these wide arrays of neurochemical messengers that can act via their putative receptors expressed on CRHNs to modulate, inhibit, or activate CRHN activity to elicit a physiological stress response during specific physiological or acute stressful conditions (psychological, physical, emotional, social and others).

Connect‐seq studies revealed subsets of upstream neurons expressed a large number and variety of neurotransmitters and neuromodulators, including fast‐acting neurotransmitters (GABA, glutamate, acetylcholine and glycine) and neuromodulators, such as biogenic amines (dopamine, histamine) (Figure 5). Consistent with the ligands expressed in upstream neurons, single‐cell transcriptome analysis revealed PVN^CRHNs^ expressed a large and diverse array of ligand‐gated ion channels, receptors for neurotransmitters (GABA, glutamate, acetylcholine, ATP and glycine), and biogenic amines (dopamine, histamine, epinephrine, norepinephrine).

The role of neurotransmitters in regulating PVN^CRHNs^ is well established. GABAergic inputs provide tonic inhibition of PVN^CRHNs^, and the application of GABA receptor antagonists readily activates PVN^CRHNs^.^83^, ^84^ Notably, GABAergic inputs might also be involved in stimulus‐induced suppression of PVN^CRHNs^ activity, as suggested by certain odours.^85^ Similarly, norepinephrine is well‐established as a stress‐induced activator of PVN^CRHNs^.^24^ Using calcium imaging, a recent study tested the direct effect of a large number of bioactive substances, including neurotransmitters, biogenic amines and neuropeptides, on PVN^CRHNs^ neuronal activity.^86^ These studies further confirmed the neurotransmitters, glutamate and acetylcholine, activated PVN^CRHNs^, whereas GABA and glycine inhibited PVN^CRHNs^. Furthermore, they have tested different biogenic amines (dopamine, serotonin, noradrenaline and histamine) and demonstrated their direct effects in rapidly activating PVN^CRHNs^.

Connect‐seq also revealed that upstream neurons remarkably expressed a large array of neuropeptides (43). While most upstream neurons coexpressed two or more signalling molecules, nearly half of the upstream neurons expressed four or more signalling molecules (up to a maximum of 10). Single‐cell transcriptome analysis of PVN^CRHNs^ revealed the expression of genes encoding GPCRs for neuropeptides and cannabinoids (Figure 5). Of the 43 neuropeptides identified by Connect‐seq in upstream neurons, a few of these have been previously shown to have a direct effect on PVN^CRHNs^ neuronal activity. Neuropeptides, such as angiotensin II (encoded by *Agt*), sulphated cholecystokinin octapeptide (CCK8S) and tetrapeptide (CCK4) (encoded by *Cck*), thyrotropin‐releasing hormone (encoded by *Trh*), significantly activated PVN^CRHNs^ and whereas nociceptin (encoded by *Pnoc*) rapidly inhibited PVN^CRHNs^. Of the other neuropeptides identified in upstream neurons, previous evidence suggests neuropeptides glucagon‐like peptide 1^87^ (encoded by *Glp1*), nesfatin‐1 (encoded by *Nucb2*) had a directly stimulatory effects on PVN^CRHNs^, whereas a few others, like oxytocin^88^ (encoded by *Oxt*) had an inhibitory effect. Connect‐seq also revealed upstream neurons expressed genes associated with known neuroendocrine functions. These include *Gnrh1* and *Kiss1*, which are master regulators of reproduction and fertility^89^; *Ghrh*, which regulates growth and metabolism^90^; and, *Trh*, which governs thyroid axis, among others.

The molecular identities identified using Connect‐seq were further used to determine the neuroanatomical locations of upstream neurons. In a different approach, molecular identities of upstream neurons were also identified using the repertoire of receptors expressed in PVN^CRHNs^. This method, termed ‘RAMUN’ (Receptor‐assisted mapping of upstream neurons), which relies on single‐cell transcriptomics of “starter cells” (PVN^CRHNs^) to identify genes encoding receptors that bind to neurochemical messengers (neurotransmitters and neuromodulators) (Figure 4). While Connect‐seq provides information on the genes expressed in upstream neurons, RAMUN relies on the gene expression detected in ‘starter’ PVN^CRHNs^. The ligand‐receptor information obtained from Connect‐seq or RAMUN can be then used to superimpose the molecular identities and determine the neuroanatomical locations of PRV‐infected upstream neurons expressing neuropeptide or neuromodulator ligands.

**FIGURE 6:**
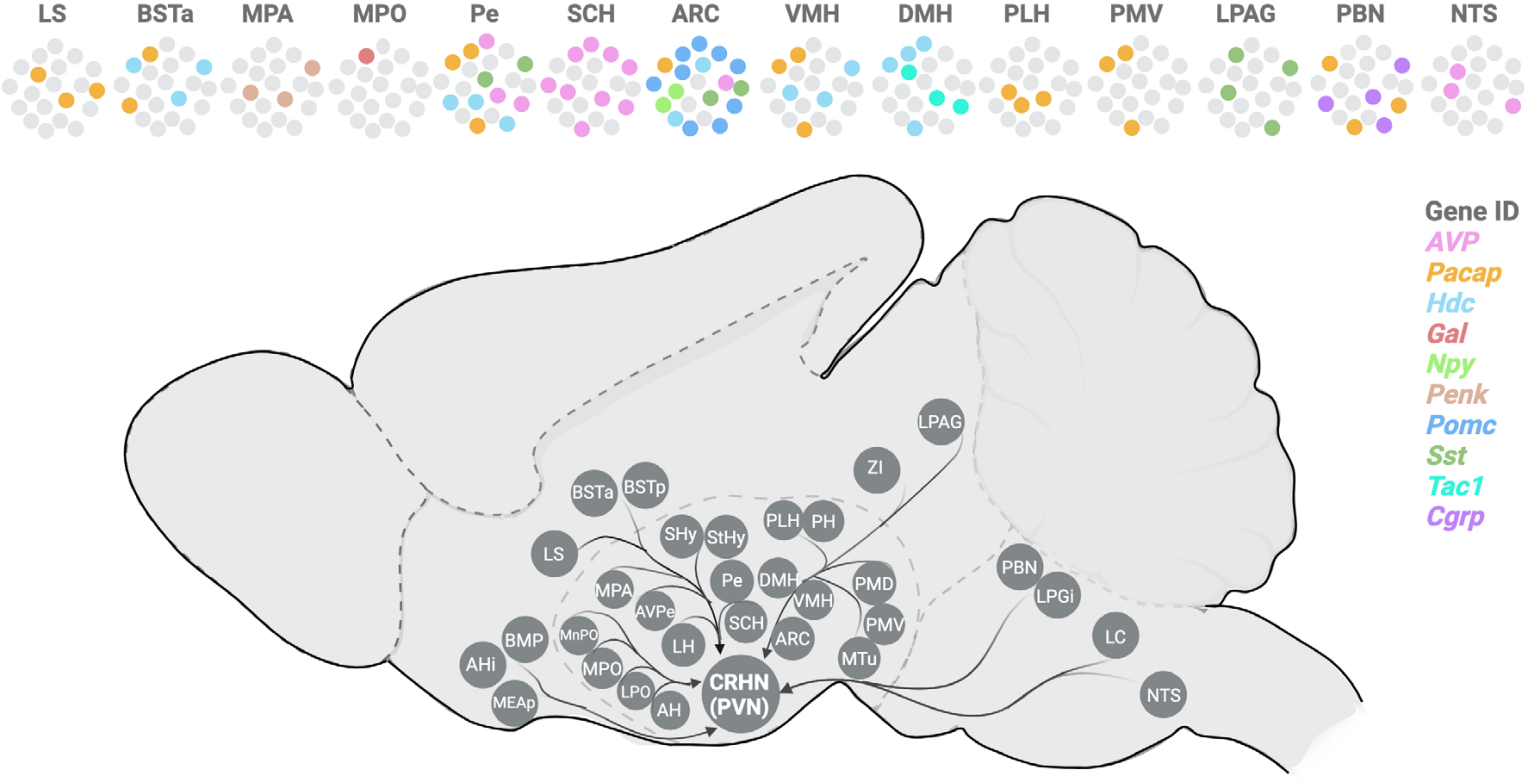
Molecular map of CRHN brain circuitry. Schematic illustrating brain areas (in circles) containing neurons that are directly upstream of CRHNs. [Top] Molecular identities of upstream neurons determined by single‐cell transcriptomics (Connect‐seq or RAMUN) and validated using dual labelling RNA‐fluorescence in situ hybridization (RNA‐FISH). Each brain area can contain upstream neurons expressing genes encoding an individual signalling molecule (LS) or two (e.g., PBN) or multiple signalling molecules (e.g., ARC). Colour of each individual cell within each brain areas indicates the gene as listed under ‘Gene ID’.

Spatial mapping of neuromodulator ligands using RNA‐FISH (flourescence in situ hybridization) revealed that upstream neurons expressed neuromodulators in specific brain areas, which can be distinguished by their anatomical locations. Altogether, 6 of 10 neuropeptides (*Pomc*, *Penk*, *Cgrp*, *Gal*, *Tac1*, *Npy*) were detected in upstream neurons in a single brain area (Figure 6). In contrast, 4 of 10 neuromodulators (*Avp*, *Pacap*, *Hdc*, *Sst*) were detected in upstream neurons in more than one brain area. It should be noted that due to the high variability of PRV infection and low number of virus‐infected cells detected per mouse brain, it is plausible that these ligands can be expressed in upstream neurons in additional brain areas.

Although Connect‐seq emerged as a powerful new tool to profile transcriptomes of circuit components, it has limitations. (A) Given that PVN^CRHNs^ are a small population of few thousands located in a tiny brain area, it can be a challenge to target these cells with high consistency between experiments. Further, it is yet challenging to target subsets of PVN^CRHNs^ that are molecularly, anatomically, and functionally distinct. This is crucial as it could lead to high variability of PRV‐infection of, both, the starter PVN^CRHNs^ and the upstream neurons. Future investigations should consider employing multiple recombinases systems (Cre, Flp and Dre) to selectively target PRVs to subpopulations of PVN^CRHNs^ co‐expressing Cre and other recombinases under the promoters of *Crh* and another marker gene of PVN^CRHNs^ (e.g., *Scgn*), respectively. It would also be interesting to employ such strategies to selectively target subpopulations of PVN^CRHNs^ that coexpress varying levels of other neuropeptides in certain physiological or chronic conditions to study the neuroplastic changes occurring due to chronic stress within the stress neurocircuitry. (B) PRVs are engineered to target genetically defined population of PVN^CRHNs^. Therefore, the number of PRV+ cells found on Day 3 post‐infection (directly upstream) per mouse brain can be extremely low (ranging from a few hundred to a few thousands).^19^ Further, due to single‐cell dissociation and isolation methods (using Flow cytometry), the yield of PRV+ cells for transcriptome analyses can be further reduced. Therefore, to generate a comprehensive molecular map of stress‐regulating brain circuitry and define their cell taxonomies, one would need a large number of cells isolated from multiple animals. Alternative methods to circumvent these challenges is to apply high‐throughput approaches can be achieved by employing single‐nucleus RNA sequencing^91^ instead of single‐cell sequencing, as it provides a better yield of PRV‐infected nuclei without compromising the number of genes and transcripts detected per cell (unpublished work). (C) Effect PRV infection on single‐cell transcriptomes: Previous studies found that neurons infected with a Bartha PRV show normal electrophysiological activity to electrical or sensory stimuli up to 48 h after infection, but abnormal activity after that,^92^, ^93^ and eventually cell death over extended periods of infection.^94^ In Connect‐seq studies, PRV+ cells were isolated on Day 3 post‐infection when presynaptic upstream neurons were infected for less than 24 h. The starter PVN^CRHNs^ infected for ~72 h likely died as none of the upstream cells analysed expressed *Crh*. Although the PRV+ upstream were infected for less than 24 h, the effects of PRV infection on their transcriptomes cannot be ruled as PRV transcripts compete with host transcripts during sequencing. Furthermore, indirect effects of PRV infection on host transcriptomes could be possible due to the activation of innate immune responses to viral infection and changes in the circulating levels of glucocorticoids due to PRV infection of PVN^CRHNs^. These limitations can be overcome by mapping PRV‐infected single‐cell transcriptomes obtained from the hypothalamus to uninfected single‐cell transcriptomes obtained from publicly available datasets.^73^, ^78^, ^95^

These studies highlight the importance of molecular mapping of neural circuits and the application of new single‐cell genomic and neurogenetic tools in understanding circuit function. For instance, a large proportion of PRV^+^ cells in the ARC expressed *Pomc*.^60^ Further, functional studies revealed Pomc^+^PRV^+^ upstream of PVN^CRHNs^ are activated by a psychological stressor, physical restraint, but not by another psychological stressor, predator odour. Chemogenetic activation of POMC neurons resulted in rapid activation of the HPA axis mimicking a stress response, and chemogenetic silencing of POMC neurons resulted in attenuated stressor (restraint)‐induced PVN^CRHNs^ activation and downstream stress response.^60^ Therefore, it would be interesting to examine how these crucial survival pathways regulate PVN^CRHNs^ function and activity, what specific signals they transmit to CRHNs and how PVN^CRHN^ circuitry may undergo pathological changes in neurodevelopmental disorders or during chronic stress.

## 8 CONCLUSIONS AND OUTLOOK

PVN^CRHNs^ represent a critical stress response pathway through which the brain regulates blood levels of stress hormones (via the HPA axis). They play a central role in integrating signals from different brain areas on a variety of internal and external dangers and help coordinate the body's responses to these stressors. In the last decade, the application of genetic tools (e.g., Cre driver lines) combined with a range of neurotechnologies (summarised below) have enabled a much refined molecular and circuit mapping of brain circuitries upstream of PVN^CRHNs^.

Using in vivo fiber photometry recordings, numerous studies have shown that PVN^CRHNs^ are rapidly activated or suppressed (within seconds of exposure) by a range of aversive and appetitive stimuli, respectively, encoding positive or negative valence.^39^ These studies indicated that PVN^CRHNs^ might be involved in regulating rapid behavioural changes in response to acute stress. Indeed, the application of optogenetics for selective photoactivation or photoinhibition revealed PVN^CRHNs^ orchestrate a complex repertoire of behaviours (increased grooming, walking, and rearing; and reduced digging and chewing)^43^ following exposure to acute stress and play an unexpected role in pheromone release and social transmission of stress,^96^ and conditioned place preference.^39^ These studies unravelled novel biological functions of PVN^CRHNs^ in stress‐responsive behavioural outcomes. These findings using advanced methods would not have been possible as prior studies have relied on IEG activation to study PVN^CRHNs^ neuronal responses to acute stressors, which have a temporal resolution of minutes to hours and provide a snapshot of past neuronal activity detected in post‐mortem tissues.

Consistent with their diverse roles in mediating stress responses, PVN^CRHNs^ receive inputs from diverse brain areas.^12^ Traditional methods have provided a detailed map of neuronal inputs to PVN from diverse brain areas, including the limbic forebrain areas (hypothalamus, amygdala, BNST and others), the brain stem and spinal cord, and circumventricular organs. Genetic approaches and sophisticated PRV‐assisted transsynaptic strategy have enabled a more detailed mapping of mono‐ and polysynaptic inputs to PVN^CRHNs^ at single‐cell resolution.^52^, ^60^ Virus‐assisted mapping revealed 31 brain areas contained directly neurons and an additional 30 brain areas that are indirectly upstream of PVN^CRHNs^. Viral tracing studies revealed findings consistent with previous studies (e.g., most hypothalamic nuclei, PBN, NTS were found directly upstream of PVN^CRHNs^). They also revealed several brain structures (e.g., MEA, LC) that are directly upstream of PVN^CRHNs^, but were previously thought to be indirectly upstream; and other brain areas (e.g., LPGi, ZI) that were previously unknown to be upstream of PVN^CRHNs^.

Viral tracing revealed a detailed map of indirect (polysynaptic) neuronal connections between the olfactory areas (five) and PVN^CRHNs^, suggesting that olfactory signals can activate or inhibit the HPA axis via PVN^CRHNs^.^52^ Indeed, IEG responses of upstream neurons in the olfactory areas and their selective manipulation of neuronal activity using chemogenetics revealed a tiny olfactory area, Ampir, is implicated in transmitting predator odour (bobcat urine and Fox faeces odour, TMT). Mapping IEG responses in directly upstream PRV‐infected neurons revealed psychological stressors, physical restraint and predator odour (TMT), are transmitted to PVN^CRHNs^ via parallel pathways.^60^ Physical restraint activated presynaptic neurons in the ARC and anterior BNST, whereas predator odour activated neurons in the anterior BNST and LPGi (hindbrain). In response to these stressors, previous studies have shown robust increases in Fos mRNA expression levels in several brain regions. In contrast, PRV‐assisted mapping of IEG responses highlights underlying circuit mechanisms through which molecularly defined subpopulations (such as POMC neurons) in specific brain areas are involved in transmitting signals to PVN^CRHNs^.

As highlighted, viral tracing methods have generated a comprehensive neuroanatomical map of direct and indirect synaptic inputs to CRHNs. Integration of viral tracing with single‐cell transcriptomics has led to the development of new tools (Connect‐seq^19^ and RAMUN^60^) to enable transcriptional profiling of upstream neurons. Consistent with earlier studies, the application of Connect‐seq revealed subsets of upstream neurons expressed genes encoding classical neurotransmitters (Glutamate, GABA and dopamine). In addition, Connect‐seq revealed upstream neurons expressed a large array of 43 different neuropeptides, and individual upstream neurons expressed multiple neuropeptides (up to 8) at different levels. Most upstream neurons expressed one or two neuropeptides at high levels and additional ones at low levels suggesting presynaptic neurons could employ the highly expressing neuropeptide for neuromodulation in basal conditions. Whereas additional neuropeptides expressed at low levels could presumably act as ‘reservoirs’ to adapt to constantly changing physiological and environmental conditions or to repeated activation during chronic stress. Analysis of single‐cell transcriptomes revealed PVN^CRHNs^ express a large repertoire of genes encoding putative receptors for neuropeptides and neuromodulators on PVN^CRHNs^, suggesting PVN^CRHNs^ have the necessary machinery to respond to constantly changing internal and external environments to rapidly integrate and maintain homeostasis.

Neuroanatomical, morphological, and circuit mapping studies indicated that the PVN^CRHNs^ are highly heterogeneous regulating neuroendocrine, behavioural, and autonomic responses to stress. Consistently, the application of single‐cell transcriptomics revealed molecularly distinguishable subsets of PVN^CRHNs^ (S*cgn*+/*Crh*+ and *Fam150b*+/*Crh*+)^72^, ^74^, ^75^ and novel mechanisms of CRH release via secretagogin and specific subpopulations impacted by early life adversities. A major limitation of these studies is that they have analysed a tiny population (~100–200) of PVN^CRHNs^, while estimates suggest a few thousand of Crh+ neurons in the PVN of rodent brains. Therefore, a deeper multi‐omics analyses of PVN^CRHN^ can provide additional insights into their neuronal and cellular functions and the mechanisms in regulating stress under basal conditions and altered physiological states.

As new technologies are rapidly advancing that enable simultaneous transcriptional and epigenomic (or multi‐omics) analyses of thousands of cells in a single experiment, and with reduced sequencing costs, it is now possible to perform high‐throughput multi‐omics analyses of circuit components and PVN^CRHNs^. The application of these technologies will not only refine the molecular heterogeneity of PVN^CRHNs^ and presynaptic neurons but also their neuronal responses to different stressors (e.g., physical vs. psychological vs. metabolic), and the underlying sex differences. Importantly, their application can shed new insights into neural circuit change in neurodevelopmental disorders (e.g., autism) or the adaptive mechanisms due to exposure to chronic stress during critical periods of development in early life or during adolescence, which have emerged as major risk factors for developing neuropsychiatric disorders. Furthermore, with recent advancements in the development of powerful new algorithms and computational tools, such as *CellPhoneDB* ^97^ or *NeuronChat*,^98^ it is now plausible to identify potential cell–cell interacting partners or infer neuron–neuron communications, respectively, from large single‐cell transcriptomics datasets.

Abbreviations for brain areas: Abbreviations used for brain areas are according to the brain mouse atlas^99^ and our previous reports.^19^, ^60^

## AUTHOR CONTRIBUTIONS

**Naresh K. Hanchate:** Conceptualization; funding acquisition; writing – original draft; writing – review and editing; visualization.

## CONFLICT OF INTEREST STATEMENT

The author declares no conflicts of interest.

## Data Availability

Data sharing is not applicable to this article as no new data were created or analyzed in this study.
